# Physiological increase of yolk testosterone level does not affect oxidative status and telomere length in gull hatchlings

**DOI:** 10.1371/journal.pone.0206503

**Published:** 2018-10-26

**Authors:** Marco Parolini, Cristina Daniela Possenti, Andrea Romano, Manuela Caprioli, Diego Rubolini, Nicola Saino

**Affiliations:** 1 Department of Environmental Science and Policy, University of Milan, Milan, Italy; 2 Department of Ecology and Evolution, University of Lausanne, Lausanne, Switzerland; University of Reunion Island, RÉUNION

## Abstract

Conditions experienced during early-life can cause the onset of oxidative stress, resulting in pervasive effects on diverse life-history traits, including lifespan. In birds, maternally-transferred egg substances may exert positive or negative influence over the offspring phenotype. Among these, testosterone can upregulate the bioavailability of certain antioxidants but simultaneously promotes the production of pro-oxidants, leading to an oxidative stress situation, which is one of the main forces causing telomere attrition However, no study has investigated the role of this androgen on telomere dynamics in birds and little is known about the effects of yolk testosterone on oxidative status in early-life of these species. We physiologically increased the levels of yolk testosterone by *in ovo* injections in yellow-legged gull (*Larus michahellis*) to evaluate the effects induced by this androgen on hatchlings plasma total antioxidant capacity, amount of pro-oxidant molecules and telomere length at hatching. Testosterone supplementation did not increase hatchling body growth, did not result in the overproduction of pro-oxidant molecules nor a reduction of antioxidant capacity. Accordingly, telomere length at hatching was not affected by testosterone treatment, although hatchlings from the third-laid eggs showed shorter telomeres than their siblings from first- and second-laid eggs, independently of testosterone treatment. Our results suggest that injection of physiological levels of testosterone does not induce oxidative stress to hatchlings and, consequently do not affect telomere dynamics during early post-natal periods.

## Introduction

The conditions experienced during early-life can result in pervasive fitness consequences through diverse physiological mechanisms, one of which involves telomere dynamics [1]. Telomeres are conserved repeated sequences of the TTAGGG nucleotide motif at the end of chromosomes that protect genomic integrity [2]. Telomeres shorten throughout life, and short telomeres at birth or rapid telomere attrition are associated with negative effects on diverse fitness traits [3,4]. Telomere attrition is more pronounced during early-life [4], possibly because intense metabolic activity underlying rapid body growth exposes the organism to the detrimental effects of oxidative stress [5]. In fact, oxidative stress, i.e. the imbalance of the equilibrium between the production of pro-oxidant molecules and antioxidant defences in favour of the former [5], has been identified as a crucial mechanism affecting telomere length [6].

In birds, mothers transfer diverse extra-genomic substances to their eggs, which exert a profound influence on offspring phenotype [7]. Whilst the allocation of exogenous antioxidants serves to protect the offspring against detrimental effects of oxidative stress [8] and can help to maintain telomere integrity during early-life periods [9], the transfer of steroid hormones can impair the oxidative status and negatively affect telomere length of the progeny [10]. Testosterone is a maternally-transferred androgen [11,12] playing a pivotal role in the regulation of embryo differentiation and development of diverse phenotypic traits [11]. As other androgens, testosterone is anabolic for muscle and skeletal growth [13–15] and supports post-natal body mass gain [16–18], although some other studies have returned none or opposite outcomes regarding its effect on post-natal growth [19–21]. Some investigations have shown that variations in the exposure to testosterone altered the oxidative balance of birds, with contrasting outcomes. For instance, high amounts of maternally transferred androgens, including testosterone, may represent a cost for offspring in terms of increased susceptibility to oxidative stress due to the accelerated metabolism [11,12]. Enhanced developmental rate mediated by androgens transferred by mothers to the eggs is associated with an increase of cell metabolism and a concomitant overproduction of reactive oxygen species (ROS) [12], with a subsequent shift of the balance between ROS and antioxidants [22]. Moreover, experimental findings have supported the idea that testosterone may also affect the antioxidant machinery [23,24]. A study of zebra finch whose egg testosterone levels were artificially elevated produced male chicks, but not female, with lower plasma antioxidant capacity [25]. Conversely, an experimental increase of testosterone levels of wild male red grouse (*Lagopus lagopus scoticus*) resulted in high levels of circulating antioxidants and high lipid peroxidation in plasma [26]. A similar manipulations of red-legged partridge (*Alectoris rufa*) produced mixed responses [27,28], while testosterone supplementation in the yolk of yellow-legged gull (*Larus michahellis*) eggs increased plasma antioxidant capacity and reduced lipid peroxidation in hatchlings during early post-natal periods [7]. Considering the capability of testosterone to affect both sides of the ‘oxidative stress’ medal, this androgen might alter the individual oxidative balance and lead to an oxidative stress situation, which can consequently change telomere dynamics. However, the effects of testosterone on telomeres have never been reported in any bird species to date, neither under captive or natural conditions. Thus, to shed light on the capability of testosterone to induce oxidative stress and to elucidate its potential contribution to telomere dynamics, we assessed the effects of a physiological increase in yolk testosterone concentration on oxidative status markers (i.e. the plasmatic amount of pro-oxidant molecules and the total antioxidant capacity) and telomere length in yellow-legged gull hatchlings. We expect that testosterone supplementation would promote the oxidative cost of growth in hatchlings, boosting the production of pro-oxidants and/or decreasing the total antioxidant capacity, and consequently reducing telomere length. In addition, we expect that telomere length would be positively correlated with plasma antioxidant capacity or negatively correlated with the concentration of pro-oxidant molecules.

## Materials and methods

### Field procedures

The present study was performed on a large breeding colony (> 400 breeding pairs) of yellow-legged gull in the Comacchio lagoon (NE Italy 44°20’ N– 12°11’ E). The colony was visited every second day to check for any new nests and newly laid eggs. When a new egg was found, it was marked to monitor the progress of laying and to identify laying sequence and temporarily removed from the nest for experimental manipulation. Removed egg was temporarily replaced with a 'dummy' egg to prevent any potential change in parental brooding behavior. Eggs were transferred to a nearby tent for manipulation and then they were brought back within two hours from the collection.

We aimed at increasing the concentration of testosterone by 1 standard deviation (SD) of the concentration measured in the yolk of yellow-legged gull eggs from the same colony [29], by the injection of a physiologically appropriate volume of a testosterone solution. As the yolk testosterone concentration in the yellow-legged gull eggs varies according to egg size and position in the laying sequence, we scaled the dose due to be injected accordingly. We grouped first- (a-), second- (b-) or third- (c-) laid eggs into three classes (i.e. tertiles) of size depending on the egg mass. Then, we calculated the standard deviation (SD) of the testosterone concentration in the yolk for each tertile within each position in the laying sequence. We estimated the yolk mass for each class size and position in laying sequence according to the following equation: yolk mass = 0.227 (0.039 SE) egg mass + 1.815 (3.461 SE); F_1,88_ = 34.38, P < 0.001). We computed the amount of testosterone due to be injected as the product of the SD (expressed in ng/g) of testosterone concentration for each tertile and position in the laying sequence and the estimated yolk mass. The injected testosterone doses were as follows (laying order: class of size according to egg mass (g), amount of testosterone (T) injected (ng per egg)): a-eggs: 84–91 g: 57 ng, 92–95 g: 59 ng, 96–108 g: 42 ng, b-eggs: 80–88 g, 74 ng, 89–92 g, 73 ng, 93–99 g: 81 ng; and c-eggs, 75–82 g, 95 ng, 82–87 g, 84 ng, 88–98 g, 76 ng. The amount of testosterone injected in the yolk of yellow-legged gull eggs was in the same range of other previous studies [7,21].

Testosterone was injected in the egg yolk according to a previously validated procedure [30]. Before the injection, the egg was weighed and placed with the longitudinal axis vertical. After eggshell disinfection, a hole close to the acute pole was drilled using a sterile pin. Injection was performed using 1-mL sterile syringe with a 0.6 × 30 mm needle, while the egg was held firmly with its longitudinal axis vertical. The hole was sealed with a drop of epoxidic glue and a small piece of eggshell superimposed to the hole soon after the injection. Testosterone solutions were prepared in sterile vials dissolving the hormone in corn oil to the final concentration required. Each vial contained the concentration of testosterone to be injected in egg yolk depending on egg mass and position in the laying sequence. We adopted a within-clutch design, whereby both control and testosterone-treated eggs were established within each clutch. We sequentially assigned the treatment schemes to the clutches, according to the order in which the first egg was found (nest, a-, b-, c-egg) as follows: nest 1, testosterone injection (T), control injection (C), T; nest 2, C-T-C; nest 3, T-C-C; nest 4, C-T-T and so forth with the following nests. Testosterone-treated eggs were injected with 30 μl of the appropriate testosterone solution, while control eggs were injected with the same volume of corn oil only.

After the *in-ovo* injection, all the nests were visited every day and eggs were monitored until hatching. Because normally up to two days elapse between the time when the egg reaches the pipping stage and hatching, we assigned chicks to their original egg injecting in the pipping egg a small drop of a food dye (either blue or green). Upon the first daily visit to the nest when any individual chick was found to have hatched, the chick was weighed (to the nearest g) and its tarsus was measured (to the nearest 0.1 mm). Finally, a blood sample (about 70 μl) was collected in heparinized capillary tubes after puncturing the hatchling ulnar vein. Blood samples were centrifuged at 11,500 rpm for 10 min to separate red blood cells from plasma, which were both stored at– 20 °C until biochemical and telomere length analyses. Molecular sexing of chicks was performed by the amplification of a section of the CHD gene [31].

### Ethics statement

This study was conducted under the permission of the Parco Regionale del Delta del Po (#252015, 20 February 2015), which allowed both the manipulation of the eggs biochemical quality and the withdrawal of a blood sample from hatchlings. Blood samples (50–100 μl) were collected by slightly puncturing the brachial vein with sterile needles and the puncturing site was carefully disinfected. No obvious negative consequences of handling hatchlings were detected.

### Methods of oxidative status markers

Total antioxidant capacity (TAC) and the amount of total oxidant status (TOS) were measured in plasma of chicks hatched from both control and T-injected eggs. TAC was measured according to a colorimetric method developed by Erel [32]. The color of 2,2’-azinobis-(3-ethylbenzothiazoline-6-sulfonic acid) radical cation (ABTS*^+^) bleaches depending on the concentration of antioxidants in the sample. The reaction is monitored spectrophotometrically and the final absorbance is inversely related to TAC of the sample. The assay was calibrated with a standard curve of Trolox and the results were expressed as μM Trolox equivalent. Mean TAC intra-assay coefficient of variation (CV) was 3.0 ± 1.2% (n = 5 replicates), while the mean inter-assay CV was 5.4 ± 3.8% (n = 3 assay plates). TOS was measured according to the colorimetric method developed by Erel [33] in order to assess the amount of pro-oxidant molecules in the sample. The oxidants in the plasma oxidize the ferrous ion-o-dianisidine complex to the ferric ion, which reacting with xylenol orange gives a blue complex. Coloration (proportional to the oxidant molecules in the plasma) was measured by a spectrophotometer at λ = 535 nm. The assay was calibrated by using a standard curve made with hydrogen peroxide (H_2_O_2_). The results were expressed as μM H_2_O_2_ equivalents. The mean TOS intra-assay CV was 3.2 ± 2.7% (n = 5 replicates) and the inter-assay CV was 5.7 ± 4.1% (n = 3 assay plates).

### Telomere length analysis

Telomere length (TL) analysis was performed according to the method described by Parolini and coauthors [34]. Genomic DNA was extracted from 10–20 μl of red blood cells using 1 mL TNSE buffer (10 mM Tris HCl, 400 mM NaCl, 100 mM EDTA and 0.6% SDS) and a standard phenol/chloroform method. We measured the quantity and purity of the extracted genomic DNA using a Nanophotometer (IMPLEN). Telomere length was measured by the monochrome multiplex quantitative PCR method (MMQPCR) using an iQ5 real-time PCR detection systems (BioRad). PCR reactions were prepared using 20 ng of genomic DNA as template, Quantitative Master Mix 2X SYBR Green (Genespin), telomere and CTCF primers at a final concentration of 1,000 nM and 500 nM each, respectively. The sequences of telomere primers for MMQPCR were (telg 5’-ACACTAAGGTTTGGGTTTGGGTTTGGGTTTGGGTTAGTGT-3’ and telc 5’-TGTTAGGTATCCCTATCCCTATCCCTATCCCTATCCCTAACA-3’), while the single copy sequence used as control was a fragment from the 12^th^ exon of the swallow CTCF gene (CCCTC-binding factor zinc finger protein). The CTCF primers used were: forward (5’-CCCGCGGCGGGCGGCGCGGGCTGGGCGGCTCCCAATGGAGACCTCAC-3’) and reverse (5’-CGCCGCGGCCCGCCGCGCCCGTCCCGCCCATCACCGGTCCATCATGC-3’). The CTCF primers are composed of a swallow genomic sequence and a GC-clamp at the 5’ end (underlined) to increase the melting temperature of the PCR product. Since the melting temperature of PCR products of telomeres and CTCF are different, both primer pairs were used in the same reaction. Cycling parameters for the PCR reactions were previously described by Parolini et al. [34] and were: Stage 1: 15 min at 95 °C; Stage 2: 2 cycles of 15 sec at 94 °C, 15 sec at 49 °C; and Stage 3: 35 cycles of 15 sec at 94 °C, 10 sec at 62 °C, 15 sec at 74 °C with signal acquisition, 10 sec at 84 °C, 15 sec at 88 °C with signal acquisition. Four-fold serial dilutions (from 10 to 100 ng) of a yellow-legged gull hatchling reference sample (DNA was extracted by blood of a coeval chick not included in the experiment) were included in each plate to produce a standard curve to measure reaction efficiency and quantify the amount of telomeric repeats and single copy gene in each sample. We used the same reference sample in each plate. All reactions were run in triplicate and six plates containing 20 samples each were performed. The testosterone and control samples were equally distributed within each plate. Five samples were replicated in each plate to assess repeatability of telomere measures. Telomere length was measured as the T/S ratio, corresponding to the ratio between the mean values of the amount of telomeric repeats (T) and of a single copy gene (S), which was then related to the T/S value of the reference sample. Thus, telomere length was expressed as relative telomere length (RTL). The mean reaction efficiencies for both CTCF and telomere amplifications were greater than 88% and 94%, respectively. In MMQPCR performed to measure relative telomere length lower efficiencies of amplification is expected compared to standard real time qPCR because the primers contain mismatches relative to a perfect TTAGGG repeat. This strategy, which is used to favor the synthesis of PCR products with homogeneous size, gives rise to lower efficiencies during the first cycles. For the control qPCR, primers containing GC clamps/adapters are used to increase melting temperature of the products allowing detection of both PCR reactions in a single well. Also in this case a relatively low reaction efficiency is expected during the first cycles. The mean intra-and inter-plate coefficient of variation (± SD) of RTL measures was 2.32 ± 2.95% and 10.76 ± 4.20%, respectively. The inter-plate coefficient of variation for RTL was not negligible and could be due to differences in qPCR efficiencies occurring among plates, affecting the RTL measurements, and enlarging the coefficient of variation of our measurements. However, considering that the effect of T treatment is far from significance, we are confident that inter-plate variability was not responsible for the absence of significant results. The intra- and inter-plate repeatability of RTL measures, expressed as intra-class correlation coefficient (ICC), was 0.68 and 0.77, respectively.

### Statistical analysis

The effect of testosterone injection on oxidative status markers and RTL was analyzed in linear mixed models (LMM), including clutch identity as a random factor. Treatment, hatchling sex and egg-laying order were included as categorical fixed-effect factors with their two-way interactions. Only non-significant (P > 0.05) interaction terms were excluded from the models in a single step. Complete models are reported in Supporting Information (S2 Table). Moreover, as oxidative stress is a driving force of telomere attrition, in order to investigate the possible covariation between RTL and oxidative status markers, we re-run the same LMM including TAC or TOS as a covariate. Complete models are reported in Supporting Information (S3 Table). Levels of TAC and TOS could not be measured in some (3–7) hatchlings because of plasma scarceness (3 samples from control group and 4 samples for testosterone treated group). We also excluded from the analyses a statistical outlier for RTL from an individual of the testosterone treated group (RTL = 0.651) after performing the Grubbs' test, also called the ESD method (extreme studentized deviate). LMM analyses were performed by SAS 9.3 PROC MIXED.

## Results

**Fig 1:**
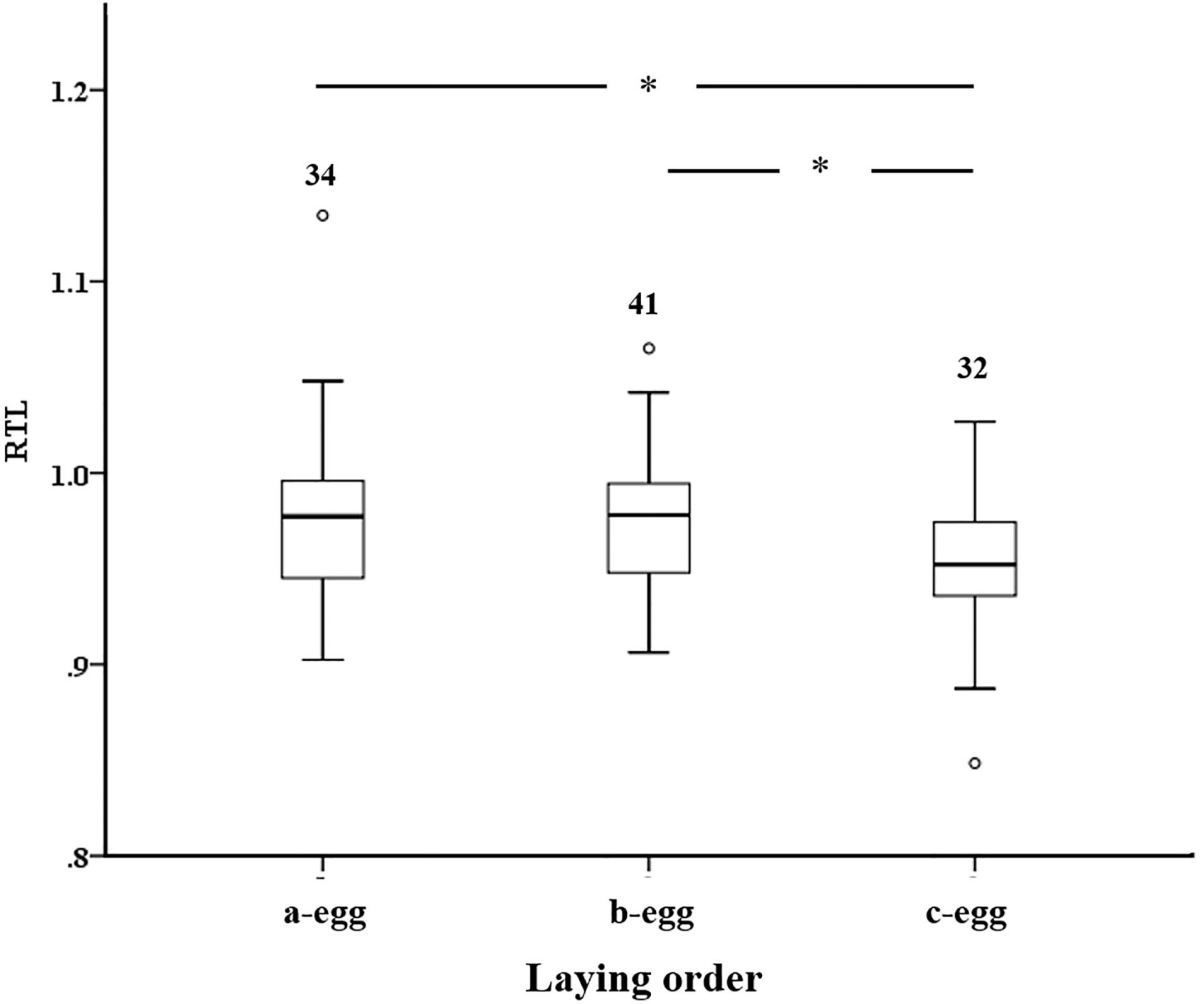
Relative telomere length at hatchling according to laying order. The number of hatchlings per each position in the laying sequence is reported.

We inoculated 201 eggs, whose hatching success significantly differed between the control (proportion of hatched eggs = 43/97 = 0.443) and T-injected (67/104 = 0.644; χ^2^_1_ = 7.387, P = 0.006) groups. The sample of hatchlings included 110 individuals from 67 clutches (mean number of hatchlings per clutch: 1.61 (0.55 SD)), with 2 clutches only (3%) containing three hatched chicks, 37 (55%) and 28 (42%) containing two and one chick, respectively. The sex ratio of hatched chicks (proportion of males) did not significantly differ between experimental groups (control eggs: 27/43 = 0.627; T-injected eggs: 28/67 = 0.418; χ^2^_1_ = 3.82, P = 0.051), although a marginally non-significant increase of females in T-treated eggs was found. At laying, egg mass did not significantly differ between the two experimental groups (F_1,49.2_ = 1.886, P = 0.176; S1 Table). However, egg mass significantly declined with the position in the laying sequence (F_2,47.2_ = 13.72, P < 0.01; estimated marginal means (SE): first-laid eggs: 88.46 (1.12) g; second-laid eggs: 87.05 (1.07) g; third-laid eggs: 82.86 (1.11) g), with significant pairwise differences between the first- and third-laid eggs, as well as between second- and third-laid eggs (LSD test; P < 0.001 in both the cases). Testosterone supplementation did not affect the incubation time (F_1,59_ = 2.46, P = 0.122), the body mass (F_1,76_ = 0.17; P = 0.683) and tarsus length (F_1,85_ = 0.02, P = 0.877) of hatchlings, after controlling for sex and laying order (S1 Fig and S1 Table). Testosterone treatment did not significantly affect neither the levels of pro-oxidant molecules nor the total antioxidant capacity in models controlling for laying order and sex effects (Table 1 and S2 Table). However, the amount of pro-oxidants depended on laying order, with hatchlings from second-laid eggs having smaller values than those from first- (P = 0.020) and third-laid (P = 0.036) eggs. Testosterone treatment did not significantly affect RTL (Table 1 and S2 Table), although it was found to vary with laying order (Fig 1), with RTL from last-laid eggs being smaller than RTL from first- (P = 0.014) or second-laid eggs (P = 0.017). Separate LMM of RTL including TAC or TOS as a covariate returned qualitatively similar results compared to original models (S3 Table) and did not reveal any significant covariation between RTL and TAC or TOS (F < 1.70 P > 0.197 in both the cases; S3 Table).

**Table 1:** Linear mixed models of total antioxidant capacity (TAC), amount of pro-oxidant molecules (TOS) and relative telomere length (RTL) in blood of yellow-legged gull hatchlings in relation to testosterone treatment, sex, and laying order. Clutch identity was included in the models as a random intercept effect. The non-significant effects of the two-way interactions between fixed factors were excluded from the final model. C = control; T = testosterone-injected (the amount of hatchling per each experimental group is reported in brackets). Significant effects are reported in bold.

	F	d.f.	P	Estimated Marginal Means (ES)
**TAC** (C = 42; T = 65)				
Treatment	0.07	1,60	0.793	C: 5,414.44 (207.48) T: 5,473.94 (174.83)
Sex	0.05	1,87	0.828	Males: 5,415.30 (198.90) Females: 5,472.08 (203.78)
Laying order	0.05	2,54	0.955	a-egg: 5,479.78 (217.76) b-egg: 5,403.28 (205.79) c-egg: 5,448.02 (222.57)
**TOS** (C = 40; T = 63)				
Treatment	0.66	1,64	0.421	C: 773.98 (93.11) T: 692.29 (79.35)
Sex	0.10	1,88	0.757	Males: 715.14 (90.58) Females: 751.13 (91.62)
Laying order	3.67	2,60	**0.031**	a-egg: 839.22 (98.64) b-egg: 564.83 (91.41) c-egg: 795.35 (99.23)
**RTL** (C = 43; T = 64)				
Treatment	0.92	1,76	0.340	C: 0.974 (0.007) T: 0.966 (0.006)
Sex	0.95	1,97	0.333	Males: 0.975 (0.006) Females: 0.966 (0.007)
Laying order	4.33	2,64	**0.017**	a-egg 0.979 (0.007) b-egg: 0.978 (0.007) c-egg: 0.953 (0.007)

## Discussion

Our results showed that an experimental increase of physiologically-relevant yolk testosterone levels did not cause an overproduction of pro-oxidant molecules nor changes in the antioxidant capacity of hatchlings. Accordingly, telomere length was not affected by the experimental treatment. However, regardless of testosterone injection, we found that telomere length decreased with the position in the laying sequence, with chicks hatched from the last-laid eggs having shorter telomeres compared to their siblings.

During early-life development, avian embryos experience a rapid increase in body mass, which is related to an increase in metabolic rate [35,36] and, consequently to the onset of an oxidative stress situation [11,12]. Some studies postulated that high amounts of maternally transferred androgens, including testosterone, may represent a cost for offspring in terms of increased susceptibility to oxidative stress. In fact, the enhancement of the developmental rate mediated by maternally-transferred androgens boosts cell metabolism and causes the consequent overproduction of ROS, which are produced during normal metabolic processes and can cause severe toxic effects to cellular macromolecules. Moreover, the risk of oxidative stress increases as embryonic growth proceeds because of the higher oxygen diffusion through the shell to support metabolism. In addition, the exposure to high concentrations of atmospheric oxygen at hatching as a consequence of the transition from a chorioallantoic to a pulmonary respiration causes oxidative stress [37]. Thus, the pre- and early postnatal conditions mentioned above can contribute to an excess of pro-oxidants that cannot efficiently be counteracted by antioxidant defenses and repair mechanisms. This would ultimately leads to an imbalance of the hatchling oxidative status and, consequently, to telomere loss [38].

### Effects of testosterone on body mass and offspring sex ratio

Contrary to our expectations, testosterone supplementation did not boost somatic growth of hatchlings. Opposite results were obtained by a previous companion study of the yellow-legged gull investigating the effects of a physiological increase of yolk testosterone levels on the phenotype of developing embryos [39], showing that embryos from testosterone-treated eggs had a larger body size and a smaller amount of residual yolk mass compared to controls [39]. These previous findings suggest that testosterone (1) enhances body size, accelerating yolk absorption and/or (2) possesses anabolic effects that are already expressed during late embryonic stages [39]. Moreover, in a previous manipulative experiment of egg testosterone levels performed on the same yellow-legged gull colony, a negative effect of testosterone was found on body mass four days after hatching [21]. Thus, the positive effect of testosterone on pre-natal body size seems to vanish at birth and turns into negative during post-natal growth. These findings are consistent with a previous study of the spotless starling (*Sturnus unicolor*), showing stronger effects of testosterone treatment during embryo development with respect to the nestling period [40]. Interestingly, T-treatment induced a marginally non-significant deviation of hatchling sex ratio towards females. These findings were partly in agreement with previous studies of birds that returned contrasting results. Whilst long-term implants of testosterone produced more male offspring in the homing pigeon [41] and spotless starling [42], and short-term injection increased the number of males in the zebra finch [43] and the white leghorn chicken [44], a correlative study of the Japanese quail showed that high circulating levels of testosterone produced more female offspring [45]. Thus, these studies showed that testosterone has the potential to mediate offspring sex ratios under natural conditions, although the mechanism by which the adjustment in sex ratio occurred in these species remains unclear.

### Effects of testosterone on oxidative status and telomere length

Conversely to findings on embryos that showed a testosterone-induced overproduction of pro-oxidant molecules in the brain and the liver [39], the supplementation of yolk testosterone did not boost the production of oxidizing compounds and did not affect the total antioxidant capacity of hatchlings. These findings are consistent with a previous study showing that the experimental manipulation of yolk testosterone did not affect neither plasmatic ROS levels nor antioxidant capacity in 1-day old yellow-legged gull chicks hatched from the last-laid eggs [7]. However, the same study showed a significant enhancement of antioxidant capacity in testosterone-treated chicks at 5 and 9 days, associated with a decrease of lipid peroxidation, suggesting that yolk testosterone may induce a mobilization of antioxidants during development and/or produce a stress during early developmental periods that could promote a compensatory response later in life [46]. Thus, a pre-natal physiological increase of yolk testosterone did not cause an oxidative stress situation to hatchlings, which consequently did not affect telomere length in our study. However, since in the yellow-legged gull the consequences of testosterone supplementation on the oxidative status emerged in late post-natal period [7], we cannot exclude an effect on telomere length in later life stages. For instance, a positive effect of a subcutaneous injection with vitamin E and methionine on telomere length was found in blue tit nestlings only one year after the treatment [40].

### Variation of telomere length according to the position in the laying sequence

Regardless of testosterone treatment, hatchling telomere length decreased with the position in the laying sequence (Fig 1). In the yellow-legged gull there is a clear laying order in the eggs produced within a clutch, with a decrease in egg size and changes in the egg composition (i.e. antioxidant and hormonal content) within broods [29]. The shortening of telomere length with laying order suggests that, at least during early-life periods, last-hatched chicks within a clutch experienced a faster rate of cellular senescence than their siblings [47]. Because in the yellow-legged gull the amount of maternally-transferred antioxidants decreases with the laying order [29], shorter telomere length of last-hatched chicks may be due to the lower amount of antioxidants compared to their siblings from first- and second-laid eggs, which do not efficiently safeguard their telomere integrity. These results are consistent with previous studies of passerine birds, showing a decrease in early postnatal telomere length between the first- and the last-hatched nestlings [47,48]. For instance, maternally-derived antioxidants such as vitamins or carotenoids decline with laying order in the zebra finch [49]. As the antioxidants play a crucial role in protecting embryos from oxidative stress during prenatal development [50,51], it is plausible that embryos from the last-laid eggs suffer high levels of oxidative stress during development, resulting in shorter telomeres compared to their siblings. Lastly, although later embryos within a clutch has higher levels of maternally-transferred testosterone [29] that might contribute to imbalance the oxidative status and affect telomere length, the present study revealed that this androgen does not contribute to the onset of an oxidative stress situation and to the consequent telomere loss at birth.

Although our investigation did not show any significant effect of a physiological increase of yolk testosterone levels, some previous experimental studies have demonstrated that maternally-transferred androgens can affect various traits of offspring phenotype, from embryonic stage to adulthood (reviewed in [52]). Considering the contrasting outcomes returned by manipulative studies of testosterone levels, an unequivocal interpretation of the effect due to maternally-transferred testosterone is difficult to be formulated [12,52–54]. The inconsistency of results might be due to differences in the experimental approach used to modulate yolk testosterone levels, which can be performed by manipulating mothers or their eggs [54]. Whilst the manipulation of mothers prevents risk of damaging the egg or affect embryo development, this experimental approach returns some shortcomings [53,54] that can complicate the interpretation of the results. Podmokła and coauthors [52] showed that the manipulation of androgen levels in the mothers did not significantly affect physiology, reproduction, survival and maternal traits of bird species because the female might limit the transfer of circulating steroids to the eggs through diverse mechanisms (e.g., selective uptake of hormones into the oocyte or by barriers to passive diffusion [55]. In addition, the maternal manipulations of testosterone can affect not only the quantity of this focal androgen transferred to the egg, but also egg and/or maternal quality [53,55]. For instance, androgens and antioxidants (i.e. vitamin E) are co-adjusted within bird eggs [56]. Thus, the administration of testosterone to the mother could result in an increased allocation of antioxidants, which might prevent or limit the direct and indirect effects due to prenatal T exposure on oxidative stress [56], and consequently on telomere dynamics. For these reasons, the injection of known amounts of hormone into the egg appears to be more appropriate method to be used in order to investigate the effects of hormones on offspring phenotype [52–54] regardless some shortcomings, namely the selection of the appropriate dose due to be injected, the choice of the solvent to be used as vehicle of the hormone and the timing of egg manipulation [52]. Thus, to investigate the possible effects of testosterone on hatchling growth, oxidative status and telomere length, we strived to prevent such limitations by 1) adjusting the testosterone dose due to be injected into the yolk according to the levels naturally occurring in the eggs of yellow-legged gull breeding in the same colony visited in the present study [29]; 2) using a lipophilic, non-toxic [20,34,39] solvent (i.e. corn oil) allowing to completely solubilize testosterone and to prevent differential distribution and bioavailability of the hormone in the yolk; and 3) manipulating the levels of testosterone at the time of laying, before the developing embryo starts producing its own hormones, in order to mimic the natural situation in which only maternal hormones occur in the egg. Thus, the lack of significant effects due to testosterone treatment cannot be ascribed to the experimental design we used. However, a recent meta-analysis [52] highlighted that the dose injected into the eggs represents the crucial factor accounting for the effects due to testosterone on offspring phenotype. Whilst no effect of solvent used, time of manipulation and site of egg injection was pointed out, the effect due to androgen (mainly testosterone) supplementation greatly depended on the injected dose. In fact, several studies highlighted a significant effect on diverse phenotypic traits after injecting a dose exceeding the natural testosterone concentration into the yolk by a couple of standard deviations, or a pharmacological dose (>5 SD up to 2,000 SD). In contrast, other studies injecting very low doses (≤ 1 SD) returned no effects due to hormonal manipulation [52], accordingly to our results. Thus, we suppose that the lack of significant effects on offspring body growth, oxidative status and consequently telomere length could be simply due to the low amount of testosterone we injected into the egg yolk. At the same time, we cannot exclude that a larger supplementation of testosterone into the yolk results in a higher growth rate and in an unbalance of offspring oxidative status due to ROS overproduction, causing telomere shortening.

In conclusion, our study showed that a physiological increase in testosterone yolk concentration has no effects on plasma oxidative status and telomere length on hatchlings. However, considering the contrasting outcomes from previous studies investigating the effects of testosterone on birds, testosterone-mediated effects may enforce trade-offs in body size and oxidative status at different ontogenetic stages (i.e. embryonic and postnatal periods). Thus, we cannot exclude that testosterone may affect body growth and imbalance the oxidative equilibrium affecting the telomere integrity in later postnatal stages, resulting in long-term consequences to the offspring.

## Supporting information

S1 Data The file summarizes all the relevant data that have been used in the statistical analyses. (XLSX)

S1 Fig Mean (±SD) of body mass and tarsus length of hatchlings according to the position in the laying sequence. Body mass was expressed in grams, while tarsus length in millimeters.
(TIF)

S1 Table Linear mixed models of egg mass at laying and incubation time in relation to testosterone treatment, sex, and laying order. Clutch identity was included in the model as a random intercept effect. The non-significant effects of the two-way interactions between fixed factors were excluded from the final model. C = control; T = testosterone-injected. Significant effects are reported in bold.
(DOCX)

S2 Table Linear mixed models of total antioxidant capacity (TAC), amount of pro-oxidant molecules (TOS) and relative telomere length (RTL) in blood of yellow-legged gull hatchlings in relation to testosterone treatment, sex, and laying order. Clutch identity was included in the model as a random intercept effect. The non-significant effects of the two-way interactions between fixed factors were excluded from the final model. C = control; T = testosterone-injected. Significant effects are reported in bold.
(DOCX)

S3 Table Linear mixed models of relative telomere length (RTL) in blood of yellow-legged gull hatchlings in relation to testosterone treatment, sex, and laying order. Total Antioxidant Capacity (TAC) and amount of pro-oxidant molecules (TOS) were singularly included in the models as a covariate. Clutch identity was included in the model as a random intercept effect. The non-significant effects of the two-way interactions between fixed factors were excluded from the final model. Significant effects are reported in bold.
(DOCX)
