# Supplementary figures and images for: Physiological increase of yolk testosterone level does not affect oxidative status and telomere length in gull hatchlings
Source: PLoS One. 2018 Oct 26;13(10):e0206503. doi: 10.1371/journal.pone.0206503 (PMC6203383; doi:10.1371/journal.pone.0206503)

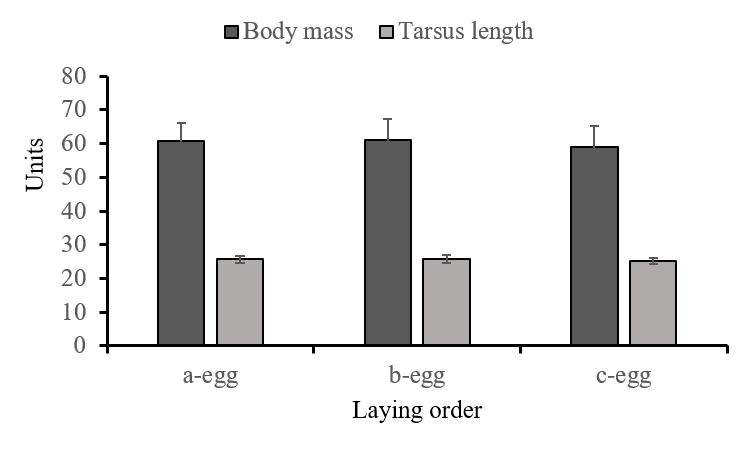

Supplement: S1 Fig — Body mass was expressed in grams, while tarsus length in millimeters. (TIF) [file pone.0206503.s002.tif]
