# Supplementary material for: Physiological increase of yolk testosterone level does not affect oxidative status and telomere length in gull hatchlings
Source: PLoS One. 2018 Oct 26;13(10):e0206503. doi: 10.1371/journal.pone.0206503 (PMC6203383; doi:10.1371/journal.pone.0206503)
Supplement: S1 Table — Clutch identity was included in the model as a random intercept effect. The non-significant effects of the two-way interactions between fixed factors were excluded from the final model. C = control; T = testosterone-injected. Significant effects are reported in bold. (DOCX) [file pone.0206503.s003.docx]

| Sample size | Egg mass  (C =42; T =65) | | | Incubation time  (C =42; T =65) | | |
| --- | --- | --- | --- | --- | --- | --- |
|  | F | d.f. | P | F | d.f. | P |
| *Final model* |  |  |  |  |  |  |
| Treatment | 1.886 | 1,49.2 | 0.176 | 3.225 | 1,57.8 | 0.078 |
| Sex | 4.592 | 1,68.1 | **0.036** | 0.046 | 1,89.6 | 0.830 |
| Laying order | 14.022 | 2,47.3 | **<0.001** | 99.943 | 2,52.8 | **<0.001** |
| *Excluded terms* |  |  |  |  |  |  |
| Treatment × sex | 1.239 | 1,58.2 | 0.270 | 0.056 | 1,74.8 | 0.814 |
| Treatment × laying order | 0.100 | 2,58.6 | 0.905 | 0.552 | 2,76.9 | 0.578 |
| Sex × laying order | 0.438 | 2,49.9 | 0.648 | 0.176 | 2,63.1 | 0.839 |
