# Supplementary material for: Physiological increase of yolk testosterone level does not affect oxidative status and telomere length in gull hatchlings
Source: PLoS One. 2018 Oct 26;13(10):e0206503. doi: 10.1371/journal.pone.0206503 (PMC6203383; doi:10.1371/journal.pone.0206503)
Supplement: S2 Table — Clutch identity was included in the model as a random intercept effect. The non-significant effects of the two-way interactions between fixed factors were excluded from the final model. C = control; T = testosterone-injected. Significant effects are reported in bold. (DOCX) [file pone.0206503.s004.docx]

| Sample size | TAC  (C =42; T =65) | | | TOS  (C =40; T =63) | | | RTL  (C =43; T = 64) | | |
| --- | --- | --- | --- | --- | --- | --- | --- | --- | --- |
|  | F | d.f. | P | F | d.f. | P | F | d.f. | P |
| *Final model* |  |  |  |  |  |  |  |  |  |
| Treatment | 0.07 | 1,60 | 0.793 | 0.66 | 1,64 | 0.421 | 0.92 | 1,76 | 0.340 |
| Sex | 0.05 | 1,87 | 0.828 | 0.10 | 1,88 | 0.757 | 0.95 | 1,97 | 0.333 |
| Laying order | 0.05 | 2,54 | 0.955 | 3.67 | 2,60 | **0.031** | 4.33 | 2,64 | **0.017** |
| *Excluded terms* |  |  |  |  |  |  |  |  |  |
| Treatment × sex | 3.00 | 1,56 | 0.088 | 0.58 | 1,74 | 0.450 | 1.26 | 1,88 | 0.265 |
| Treatment × laying order | 2.03 | 2,47 | 0.142 | 0.84 | 2,67 | 0.435 | 0.45 | 2,79 | 0.640 |
| Sex × laying order | 1.61 | 2,56 | 0.209 | 0.68 | 2,76 | 0.509 | 0.45 | 2,81 | 0.637 |
