# Supplementary material for: Physiological increase of yolk testosterone level does not affect oxidative status and telomere length in gull hatchlings
Source: PLoS One. 2018 Oct 26;13(10):e0206503. doi: 10.1371/journal.pone.0206503 (PMC6203383; doi:10.1371/journal.pone.0206503)
Supplement: S3 Table — Total Antioxidant Capacity (TAC) and amount of pro-oxidant molecules (TOS) were singularly included in the models as a covariate. Clutch identity was included in the model as a random intercept effect. The non-significant effects of the two-way interactions between fixed factors were excluded from the final model. Significant effects are reported in bold. (DOCX) [file pone.0206503.s005.docx]

|  | RTL | | |
| --- | --- | --- | --- |
|  | F | d.f. | P |
| *Final model* |  |  |  |
| Treatment | 0.973 | 1,74.2 | 0.327 |
| Sex | 0.995 | 1,95.0 | 0.321 |
| Laying order | 4.386 | 2,64.7 | **0.016** |
| TAC | 0.558 | 1,87.6 | 0.457 |
| *Excluded terms* |  |  |  |
| Treatment × sex | 1.374 | 1,86.5 | 0.244 |
| Treatment × laying order | 0.530 | 2,85.7 | 0.591 |
| Sex × laying order | 0.407 | 2,78.9 | 0.667 |
|  |  |  |  |
| *Final model* |  |  |  |
| Treatment | 0.437 | 1,70.6 | 0.511 |
| Sex | 1.317 | 1,87.4 | 0.254 |
| Laying order | 4.124 | 2,61.7 | **0.021** |
| TOS | 1.796 | 1,61.8 | 0.185 |
| *Excluded terms* |  |  |  |
| Treatment × sex | 0.711 | 1,76.6 | 0.402 |
| Treatment × laying order | 0.853 | 2,82.5 | 0.430 |
| Sex × laying order | 0.437 | 2,74.6 | 0.647 |
